# The Erie County Hospital Again

**Published:** 1896-01

**Authors:** 


**A Monthly Review of Medicine and Surgery.**

*EDITORS:*

THOMAS LOTHROP, M. D. - - WM. WARREN POTTER, M. D.

All communications, whether of a literary or business nature, should be addressed to the managing editor: 284 FRANKLIN STREET, BUFFALO, N. Y.

VOL. XXXV. JANUARY, 1896. No. 6.

### THE ERIE COUNTY HOSPITAL AGAIN.

A FEW months ago we gave our readers extended comments on the evolution of this hospital and suggestions as to its further improvement. Since that time there have been many meetings between the hospital staff and a committee of the board of supervisors, whose special province is to exercise watchful care over the almshouse and hospital.

Though these meetings of late have been spirited and interesting, during which the various aspects of the case have been set forth clearly and ably by the staff, we yet fail to observe that any impression has been made upon the county legislature that promises any reform in the conduct of the hospital. Indeed, according to the latest published newspaper reports, any expectation of relief at the hands of the present board of supervisors is very remotely dim and nebulous.

The chief bone of contention just now is the administrative separation of the hospital from the almshouse. To the casual observer this would seem to be a most commendable change. There appears to be no good reason why the keeper of the almshouse, an elective county officer subject to all the vicissitudes of politics, should exercise authority over the hospital. We know how difficult it is to make politicians yield any vantage ground, which even a poorhouse may be supposed to give them, but we cannot believe even they will long resist the better instincts of humanity when it appeals with so much justice in its cause. It has been affirmed that there is an undertow of opposition to this proposed divorcement of authority, on the part of the controlling spirits of the two other hospitals, but we cannot believe this to be true. Surely there are no men on the staff of either one of the older hospitals—

none who has any right to speak authoritatively—that would take such an unwarranted position, one in opposition to the spirit of this humane and progressive age. We should be sorry to believe this true of any members of our philanthropic and humane profession.

The matter may then be said to have settled down to a contest between the hospital staff, supported by every philanthropic and charitably disposed citizen on the one hand, and the politicians, supported by the spoils-hunters, on the other. The ultimate result of such a contest cannot be doubtful. The principle of right may be somewhat delayed in the exercise of its authority, but it must surely triumph in the end. It always has ; it always will.

It seems to us that the proper way would be to organise a city and county hospital to be supported by appropriations from the city and county treasuries, each territorial division to contribute according to the number of patients each respectively sends to be treated within its walls. Such a hospital should be governed by commissioners from each branch and should be presided over by a medical superintendent, nominated by the medical staff, and confirmed by the commissioners. These commissioners should be chosen from among the wealthiest and most philanthropic citizens residing in the city and towns. Such a hospital would be in a position to command the respect of the entire population. The almshouse itself should be removed to a more rural location.

Cincinnati, a city close to Buffalo in size, maintains a hospital at municipal expense that is one of the most successful institutions of its kind in the country. Buffalo, destined to grow rapidly in the future, with its largely increasing manufacturing interests, must necessarily provide itself with ever-increasing hospital accommodations. Trolley ambulance service, which cannot be much longer delayed, will place each and all the hospitals within easy reach. One of the prides of greater Buffalo should be its adequate hospital accommodations and its electric railway ambulances.

